# Structural Insights into Putative Molybdenum Cofactor Biosynthesis Protein C (MoaC2) from *Mycobacterium tuberculosis H37Rv*

**DOI:** 10.1371/journal.pone.0058333

**Published:** 2013-03-19

**Authors:** Vijay Kumar Srivastava, Shubra Srivastava, Ashish Arora, J. Venkatesh Pratap

**Affiliations:** Molecular and Structural Biology Division, CSIR-Central Drug Research Institute, Mahatma Gandhi Marg, Lucknow, India; Indian Institute of Science, India

## Abstract

The Molybdenum cofactor (Moco) biosynthesis pathway is an evolutionary conserved pathway seen in almost all eukaryotes including the pathogenic species *Mycobacterium tuberculosis.* This pathway comprises of several novel reactions which include the initial formation of precursor Z from guanosine triphosphate (GTP), catalysed by two enzymes MoaA and MoaC. Although Moco biosynthesis is well understood, the first step is still not clear. In *M. tuberculosis H37Rv*, three orthologous genes of MoaC have been annotated: moaC1 (Rv3111), moaC2 (Rv0864) and moaC3 (Rv3324c). Rv0864 (MoaC2) is a 17.5 kDa protein and is reported to be down-regulated by ∼3 times in the nutrient starvation model for *Mycobacterium tuberculosis.* The crystal structure of Moco-biosynthesis protein MoaC2 from *Mycobacterium tuberculosis* (2.20 Å resolution, space group *P*2_1_3) has been determined. Based on a comparative analysis of structures of homologous proteins, conserved residues were identified and are implicated in structural and functional roles. Molecular docking studies with probable ligands carried out in order to identify its ligand, suggests that pteridinebenzomonophosphate as the most likely ligand. Sequence based interaction study identified MoaA1 to interact with MoaC2. A homology model of MoaA1 was then complexed with MoaC2 and protein–protein interactions are also discussed.

## Introduction

**Figure 1:**
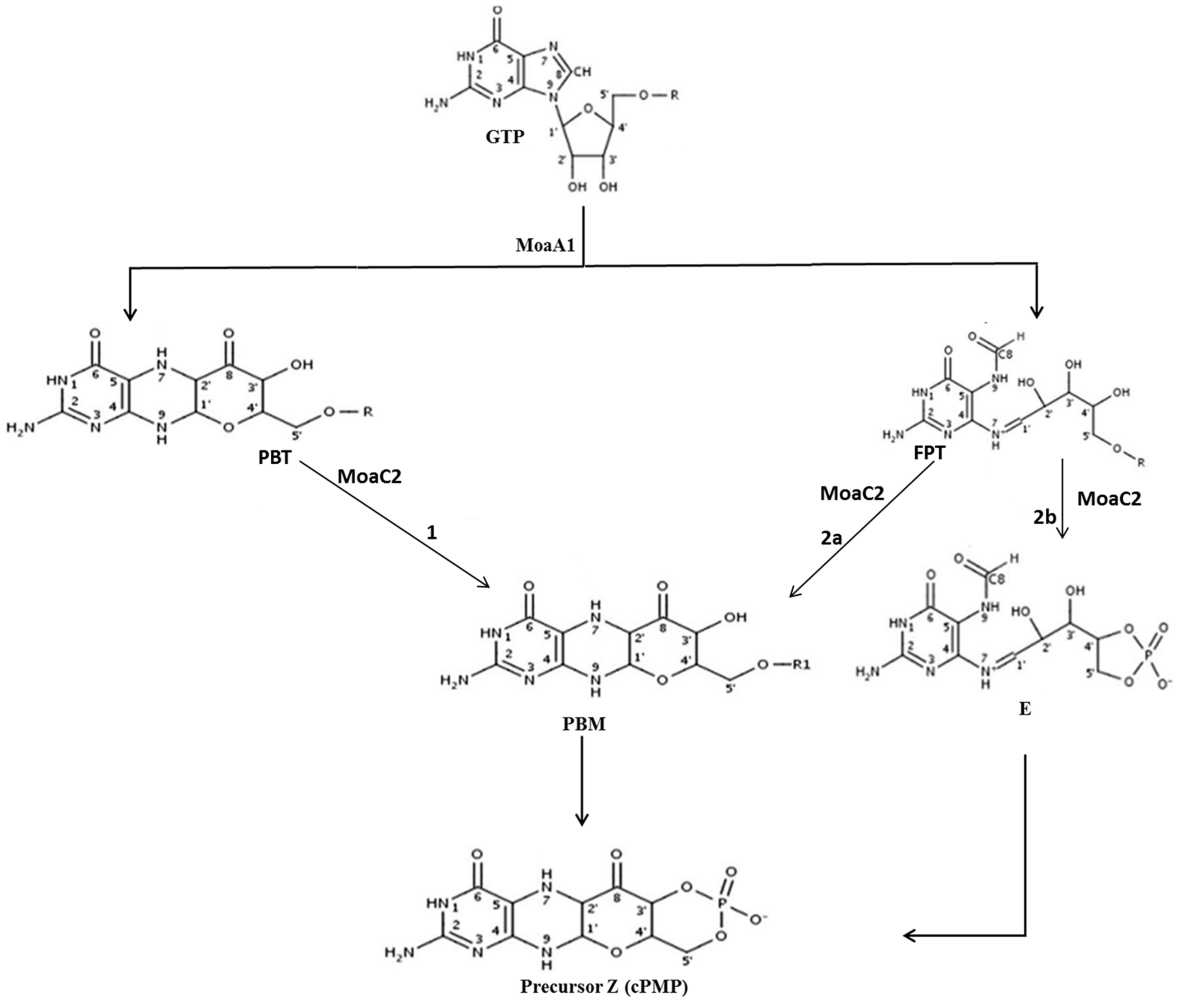
Schematic representation of the possible mechanisms proposed for the first step of the Moco biosynthesis pathway involving two probable substrate molecules FPT and PBT for MoaC. R and R1 denote triphosphate and monophosphate groups, respectively [8].

Rv0864 (MoaC2) from *Mycobacterium tuberculosis* is one of the enzymes in the molybdenum cofactor (Moco) biosynthesis pathway. Together with MoaA, MoaC is involved in the conversion of guanosine triphosphate (GTP) to precursor Z, the first step in Moco synthesis [1], [2]. Details of the Moco biosynthesis pathway, including the types of molybdenum cofactors and genetic deficiencies caused due to Moco biosynthetic enzymes, have been discussed in Srivastava *et al*., 2012 [3]. MoaA belongs to the S-adenosylmethionine (SAM)-dependent radical enzyme superfamily, members of which catalyse the formation of protein or substrate radicals by reductive cleavage of SAM by a [4Fe– 4S] cluster [4], [5], [6]. Although moco biosynthesis is well understood, its first step, the synthesis of precursor Z from GTP, is still not clear. It is thought that the GTP molecule first binds to MoaA and an intermediate compound (formamidopyrimidine-type; FPT) is generated which is subsequently used by MoaC. MoaC catalyzes the release of pyrophosphate from FPT and the formation of the cyclic phosphate of precursor Z [7]. Kanaujia *et al*., (2010) [8] proposed that the formation of precursor Z can occur in two possible pathways: in the first path where FPT (formamidopyrimidine-type) is the substrate of MoaC [7] and precursor Z is formed either via the formation of intermediate compound E (formamido-type) or PBM (pteridinebenzomonophosphate), and in the second case, compound PBT (pteridinebenzotriphosphate) may play the role of the substrate of MoaC which is formed when the ring formation of FPT molecule is completed. In this pathway, PBM is the intermediate formed before precursor Z (Figure 1).

At present, it is also unclear whether MoaA and MoaC act independently of each other or form a protein-protein complex. In humans, the molybdenum cofactor synthesis step 1 (MOCS1) locus has been reported to produce two enzymes (MOCS1A and MOCS1B) from non-overlapping ORFs within a bicistronic transcript [9]. The functional characterization of the human proteins MOCS1A and MOCS1B as well as the MOCS1A-MOCS1B fusion protein that catalyze the formation of precursor Z have been identified [9], [10], [11]. MOCS1A (MoaAs) from eubacteria and eukaryotes contain a functionally important double Gly motif at the C-terminus [12]. A likely function of this double glycine motif in MOCS1A might be the interaction with MOCS1B, forming a stable MOCS1A-MOCS1B protein complex or a transient complex during catalysis, as in the MoaD-MoaE and MoeB-MoaD protein complexes [13], [14]. The C-terminal loop of MOCS1A might interact with the active site of MOCS1B, which is formed by two monomers in the homologous MoaC protein from *E. coli* [15].

In the Betts *et al*., 2002 [16] starvation model, mimicking *Mtb*, many genes appear to be down-regulated, including those that are involved in energy metabolism, lipid biosynthesis, cell division, and transcription. In contrast, starvation induces a number of stress response and virulence factors that may be important for adaptation to the host environment. In this model MoaC2 is reported to be down-regulated by ∼3 times after 4 hour starvation. Precursor Z is one of the most stable intermediates in the Moco pathway having a half-life of several hours at low pH [17]. In addition to these orthologous gene moaC1 (Rv3111) is necessary for intermediary metabolism. In primate model, mutation in this particular gene of *Mycobacterium tuberculosis* causes attenuation therefore the mechanism of Moco pthaway is necessary to understand which is still not clear [18].

Here we report the crystal structure of MoaC2 from *Mycobacterium tuberculosis H37Rv.* The cloning, expression, purification and crystallization were already discussed in Srivastava *et al*., 2012 [3]. As our attempts to crystallize the protein with guanosine triphosphate (GTP) or other ligands have been unsuccessful, computational docking studies were initiated with the probable ligands from the pathways proposed by Kanaujia *et al.*, 2010 [8] in the formation of precursor Z. In addition sequence based interaction study was done to know if MoaC2 and MoaA interact with each other and further protein-protein docking was done with identified interacting partner.

## Results and Discussion

### The Structure of MoaC2

**Figure 2:**
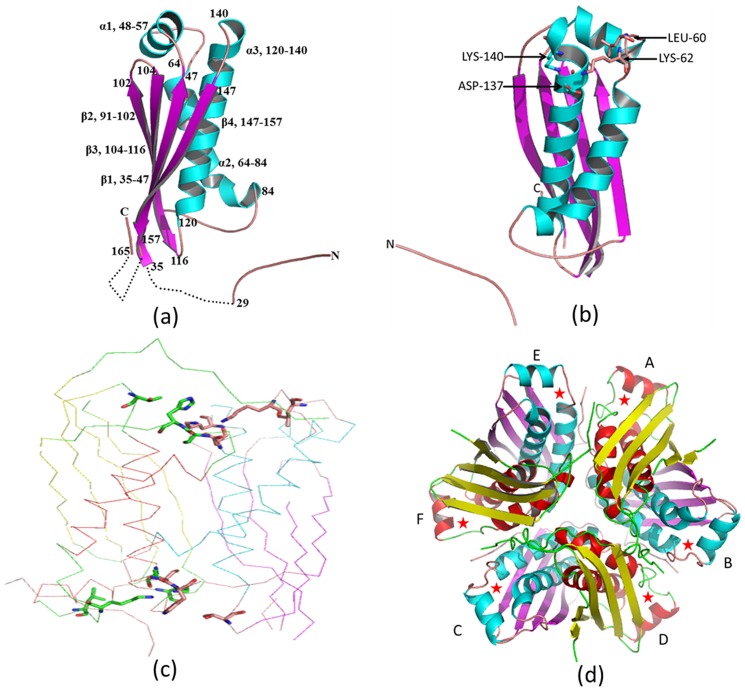
Tertiary and Quaternary structures of MoaC2 (Rv0864). Cartoon representation of the (**a**) monomer; helices are coloured in cyan and sheets in magenta and loop regions are in salmon. Missing regions are shown as dots. (**b**) Monomer showing the residues of the putative ligand binding site as sticks. (**c**) C-α trace of contents of the asymmetric unit (dimer) with the residues of the putative ligand binding sites shown as sticks.(**d**) hexamer the ligand binding site are shown as asterisks.

The MoaC2 monomer has a common α+β protein fold that is composed of a four stranded anti-parallel β-sheet and three helices, α1, α 2 and α3 located underneath the β-sheet (Figure 2 a). The fold of MoaC belongs to the ferredoxin like family (βαββαβ) with one additional helix (βααββαβ) not typically present in a prototypical ferredoxin-like molecule. The additional helix (α1) which is not typically present in ferredoxin like fold, may impart functional role. The asymmetric unit of Rv0864 (MoaC2) contains a dimer and three such dimers related by crystallographic symmetry associate to form a hexamer, which is the biological assembly. The two independent subunits in the structure superpose onto each other with a root mean square deviation (r.m.s.d) of 0.35 Å for 120 C*α* atoms. Closer examination of the superposition reveals conformational differences in the additional helix α1, wherein the r.m.s.d between α1 region of subunit A and B is 0.46 Å. Upon dimerization, the 4-stranded anti parallel beta sheets of both monomers associate to form a contiguous 8 stranded sheet. The α3 helix is present at the dimer interface of MoaC2, α2 helix, loop region residues 83–89 (LIPLCHQ) and Leu122 and Ile148 are mainly involved in the dimerization of the protein molecule. The surface areas buried at the intra-dimeric and inter-dimeric interfaces in the hexamer are given in Table 1. The intra-dimeric interface area is greater than the other interface. Also, while 14 hydrogen bonds stabilize this interface, while eight hydrogen bonds are observed in the other interface. From the surface area calculations and number of inter subunit hydrogen bonds at the interfaces, it can be speculated that MoaC2 exists as a trimer of dimers wherein the dimer counterparts are held together by hydrophobic and hydrophilic interaction. During oligomerization, a total of 21% and 42% area of monomeric units was buried upon dimerization and hexamer formation. The solvation free energy gain upon formation of dimer and hexamer assembly, calculated using the *PISA* webserver [19] are −25 kcal mol ^−1^ and −106.9 kcal mol ^−1^ which suggests that hexamer assembly is more stable.

**Table 1:** Buried surface area and hydrogen bonds at the intra-dimer and the inter-dimeric interfaces in hexamer .

Chains	No. of interface residues	Interface area Å^2^	No. of hydrogen bonds
Intra-dimer	35∶34	1586∶1607	14
Inter-dimer	14∶16	727∶734	8

The structure of MoaC2 was solved in its native form, without any ligand. The putative binding sites, as identified from a superposition with the homologous *T. thermophilus* structure (PDB ID 3JQM) [8] are shown as stick in monomer, dimer and asterisk in hexamer (Figure 2 a, b, c).

### Comparison of Rv0864 (MoaC2) with its homologues from other sources

Structural information on MoaC is available from *apo* crystal structures from *Escherichia coli* (EcMoaC; PDB entry 1ekr) [5], *Pyrococcus horikoshii* (PhMoaC; PDB entry 2ekn, *Sulfolobus tokodaii* (StMoaC; PDB entry 2ohd [20], *Geobacillus kaustophilus* (GkMoaC; PDB entry 2eey and GTP bound from *Thermus thermophilus* (TtMoaC PDB entry 3JQM) [8] are available.

**Figure 3:**
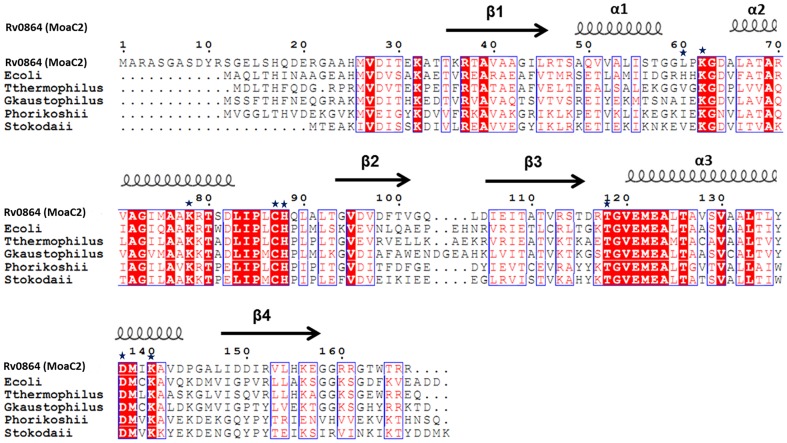
Structure based Sequence alignment of Rv0864 (MoaC2) with different MoaC proteins. Helices and strands are represented by coils and arrows respectively. Conserved residues are highlighted in red boxes. Residues of the ligand binding sites are marked with asterisks. The sequence alignment was produced using the program *ClustalW* [46] and the figure generated using *ESPript 2.2* [47].

A structural based sequence alignment of Rv0864 with *E.coli*, *T.thermophilus*, *G. kaustophilus*, *P.horikoshii and S.tokodaii* is shown in Figure 3. The overall sequence identity of Rv0864 with all other MoaC proteins is 17% though the pairwise sequence identity of these proteins with Rv0864 are higher (∼44% with *E.coli*, *T.thermophilus*, *G.kaustophilus*, *P.horikoshii* and with ∼35% with *S.tokodaii*) .

It also shows that 31 of 167 residues are conserved among the species. These conserved residues play an important functional or structural role. The Ala69, Gly73, Ala76 and Lys78 present on the α2 helix play a significant role in the oligomerization of protein the protein molecule. Lys78 which is conserved in all MoaC proteins is located in the α2 helix of the adjacent dimer forms three strong hydrogen bonds through its NZ (Average distance 2.8 Å) to the main chain carbonyl group of residues Ser81, Pro85 and Cys87, with an average distance of ∼2.8 Å resulting in an approximately three fold symmetrical arrangement.

**Figure 4:**
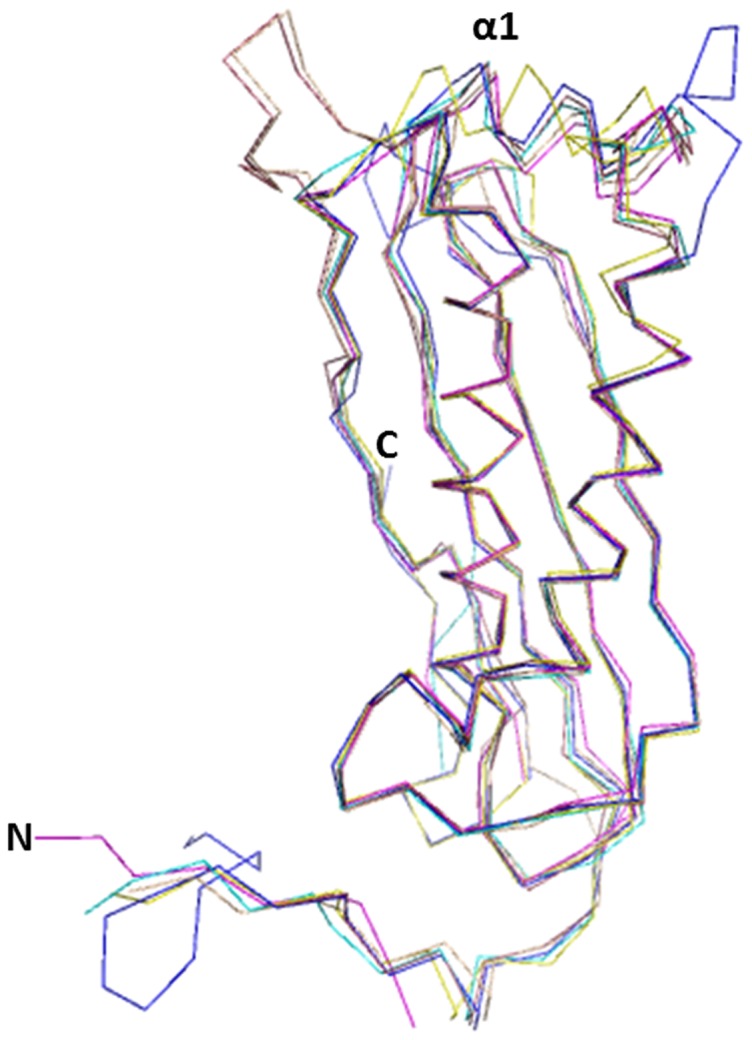
C-α trace of the structural superposition of the individual subunit of Rv0864 MoaC2 (magenta) with those of TtMoaC (yellow), EcMoaC (cyan), PhMoaC (wheat), StMoaC (raspberry) and GkMoaC (blue). The figure was generated using *PyMOL* (v.1.2r3pre; Schrodinger LLC).

The tertiary structure of Rv0864 (MoaC2) is similar with *T.thermophilus*, *E.coli*, *G.kaustophilus*, *P.horikoshii* and *S.tokodaii* with root mean square deviation of 1.45, 0.96, 2.7, 1.4 and 0.98 Å respectively (Figure 4). The maximum deviation is in the α1 region (Table S1). Each dimer of Rv0864 is stabilized by 14 hydrogen bonds as compared to eleven in *T.thermophilus* Moac [8] and eight hydrogen bonds in the *E.coli* MoaC [15].

**Figure 5:**
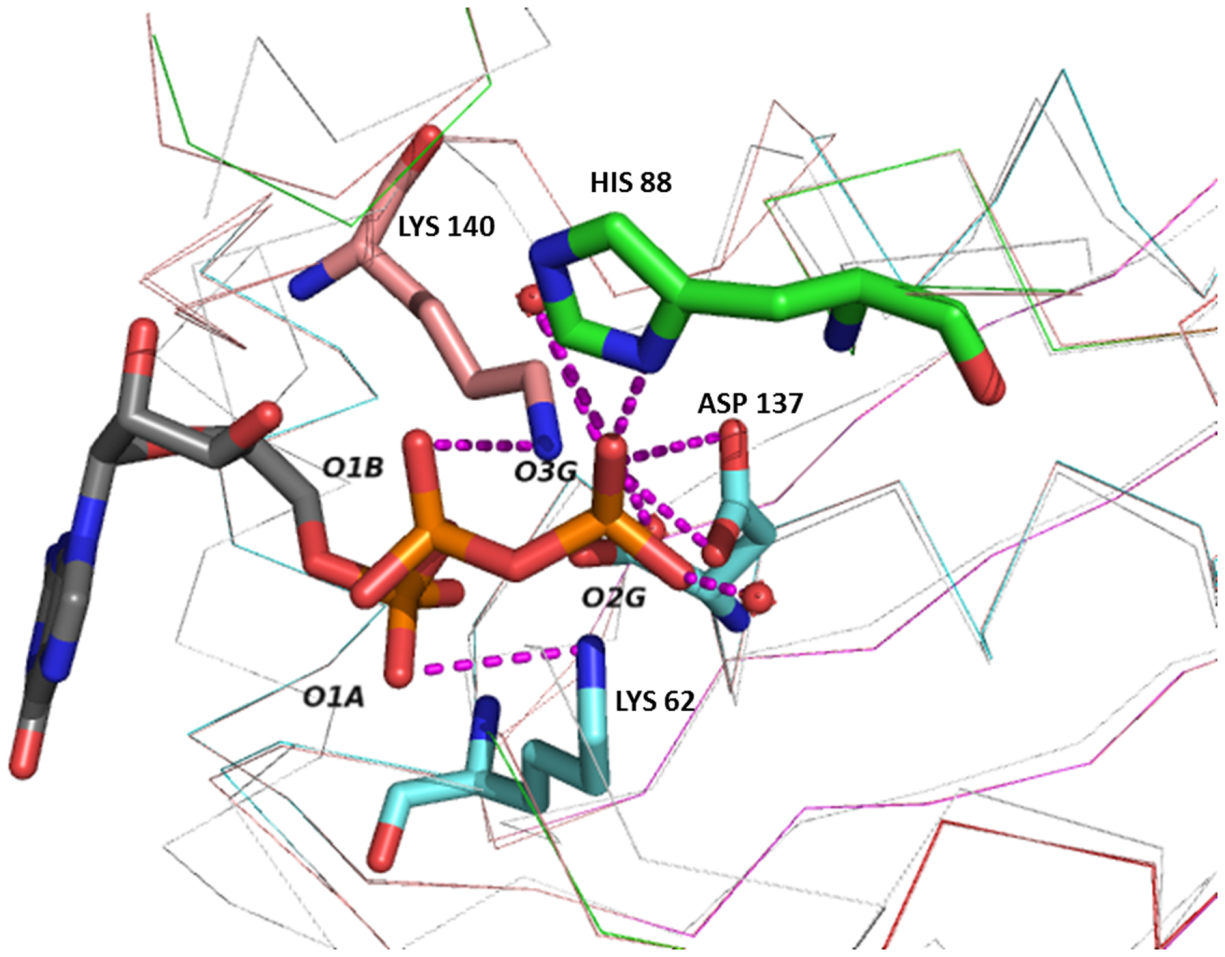
The putative ligand binding site of Rv0864 with GTP, obtained from its structural superposition on TtMoaC. Water molecules are represented as red spheres. The figure was generated using *PyMOL* (v.1.2r3pre; Schrodinger LLC).

In order to know the putative ligand binding site, the coordinates of Rv0864 (MoaC2) was superposed on *T. thermophilus* [8] coordinates with a root mean square deviation of 1.45 Å. The GTP forms hydrogen bond with Lys62, Asp137, Lys140 of A chain with distance 3.4 Å, 2.1 Å, 3.1 Å and His88 of B chain with distance 2.6 Å, which are in qualitative agreement with the *T. thermophilus* structure. Additionally, three water molecules form four hydrogen bonds with GTP, two of them with an oxygen of the third phosphate O3G with distances of 2.7 Å, 2.3 Å, and the other two with O1G and O2G with distances of 2.4 Å and 2.5 Å respectively. These water mediated interactions are not observed in *T. thermophilus* structure [8] (Figure 5).

The protein MoaC2 is mainly exist in a hexameric form which, however, easily dissociates into its dimer in solution, while the functional oligomeric state is unknown [21]. We analyzed the oligomerization states of all available crystal structures of MoaC using the *PISA* web server. The result suggests that *EcMoaC*, *TtMoaC*, *StMoaC*, *PhMoaC* and *GkMoaC* are predicted to be stable in dimeric, trimeric or hexameric state,with the hexameric state being the most stable. It is interesting to note that the six MoaC structures in the PDB (including Rv0864) differ in their crystallization conditions, space groups and asymmetric content (monomer to a nonamer), yet exhibit this hexameric assembly.

### Result of Docking

As mentioned earlier, the first step in Moco biosynthesis, the formation of precursor Z from a guanosine derivative (GTP) is still unclear. It is reported that MoaA generates an intermediate compound from the guanosine derivative and subsequently MoaC acts on it to form precursor Z [7]. Our attempts to crystallize MoaC with GTP (either by co-crystallization or by soaking) have been unsuccessful. In fact, one of the native crystals soaked in 10 mM GTP diffracted to 2.8 Å, but no GTP could be seen in the electron density map (Data not shown). This seems to suggest that either MoaC2 does not bind GTP or it binds rather weakly. Therefore to identify the ligand of MoaC2, *In-silico* docking studies were done with GTP and other possible intermediates compounds, in the formation of precursor Z as given by Kanaujia *et al.*, 2010 [8]. Such structure based computational calculations provide significant mechanistic insights. Ongoing experimental work in one of groups (AA) have been initiated based on the study.

**Figure 6:**
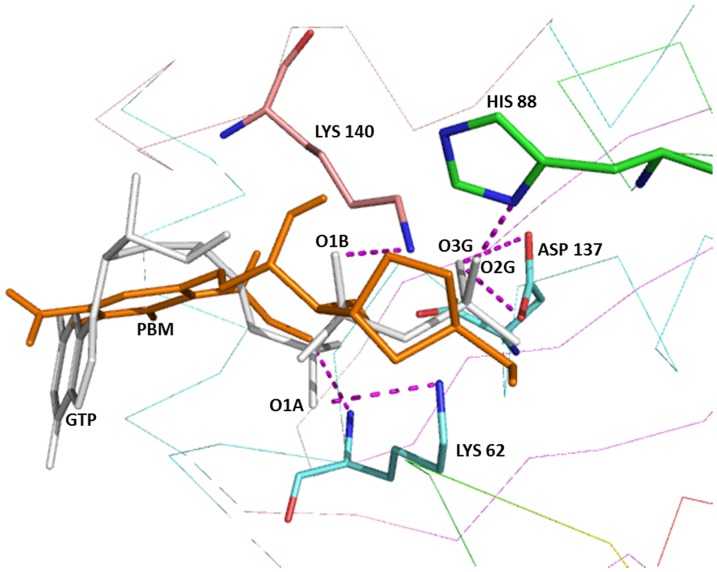
A close up view of the the ligand binding site of Rv0864 with the docked GTP and PBM, two of the possible ligands in the precursor Z formation pathway. Hydrogen bonds are represented as broken lines (magenta).

To identify the most likely ligand that binds to MoaC2, all compounds in the pathway from GTP to precursor Z (GTP, FPT, PBT, PBM, E and precursor Z) were docked to MoaC2 using *Autodock* 3.0.5 and their interaction energies calculated using its empirical scoring function. The docking of GTP shows hydrogen bonds between the oxygens of phosphates to the active site residues Lys62, Asp137, Lys140, Leu60 of A chain and His88 of B chain, in agreement with the *T. thermophilus* structure [8] (Figure 6). Table S2 lists the docking energies for these ligands and the hydrogen bonds between the protein with GTP and probable ligands. From S2, it is evident that PBM has the highest docking energy, followed by GTP. Apart from the hydrogen bonds observed between GTP and MoaC2, which are consistent with the crystal structure, PBM has an additional hydrogen bond between Lys 140 with oxygen of carbonyl group of the benzo moiety (N-O32, 2.93 Å). The hydrogen bond distances are consistently shorter in PBM approximately by ∼0.50 Å as compared to GTP. Qualitatively, this suggests that PBM is a better, and therefore more likely ligand than GTP which is also in agreement with the existing hypothesis, where MoaA acts on GTP and MoaC involved in the subsequent step of precursor Z synthesis. The docking studies also suggest that of the three pathways proposed for precursorZ formation by Kanaujia *et al.*, one of them (Path 2b, Fig. 1) can be ruled out, based on comparative interaction energies for the different ligands (S2).

### Result of sequence based interaction study

**Figure 7:**
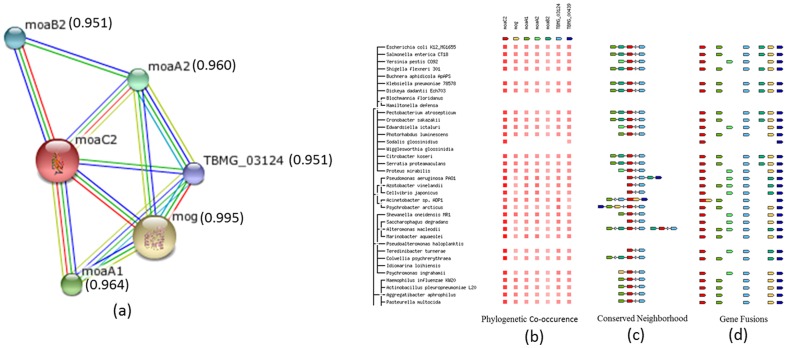
Summary of the outputs from String server with *M. tuberculosis* MoaC2 as the query molecule. (**a**) Network diagram connecting potential partner proteins. Green lines indicate association by recurring neighborhood, blue lines represent phylogenetic co-occurrence, red lines indicate gene-fusion events and light green corresponds to text mining; the thickness of each line is a rough indicator for the strength of the association. The interacting partners are mog (molybdopterin biosynthesis protein), moaA1 (molybdenum cofactor biosynthesis protein A moaA1), moaA2 (molybdenum cofactor biosynthesis protein A), moaB2 (pterin 4-alpha-carbinolamine dehydratase) and TBMG_03124 (molybdenum cofactor biosynthesis protein E2) and their confidence score are given in bracket. Contributions from (**b**) Phylogenetic co-occurrence in which a dark color shows the conservation of the gene and light color shows no homolog. (**c**) neighborhood wherein genes connected by lines are direct neighbors on the chromosome. (**d**) hybrid gene which is formed from two previously separate genes.

In order to find if moaA and moaC2 interact with each other during the formation of precursor Z, the sequence of moaC2 was given as input to the *STRING* 9.0 (http://string-db.org/) [22] to identify potential interacting proteins and its result is shown in Figure 7 a. mog and two orthologous genes of moaA, moaA1 (Rv3109) and moaA2 (Rv0869c) are identified as potential interacting partners of MoaC2 (Rv0864). mog is annotated to be responsible for the downstream step of converting MPT to MPT-AMP, while moaA1 and moaA2 are involved in the same step of Moco biosynthesis. However, neither of the MoaA structures from *M. tuberculosis* is available in the PDB. *BLAST* searches of the sequences of both MoaA1 and MoaA2, against the PDB were done, to identify homologous structures, showed a homologous structure from *Staphylococcus aureus* (PDB ID 2FB3) [7]. The *S. aureus* MoaA has a higher sequence identity with MoaA1 (37.7%) than MoaA2 (29%). Hence, MoaA1 was chosen as the representative MoaA structure for further *in-silico* Protein-Protein interaction studies, (Figure 7b–d). As mentioned earlier, the *SwissProt* server (http://swissmodel.expasy.org/; [23] was used to build the model of moaA1.

Having identified that MoaA1 and MoaC2 interact, the two sequences were then input to the *PPI-Pred* interaction server (http://bmbpcu36.leeds.ac.uk/ppi_pred/; [24] which identified and ranked three clusters of residues on both proteins as potential interacting sites. This further validates that MoaA1 and MoaC2 may be interacting with each other.

### Result of protein-protein docking

**Figure 8:**
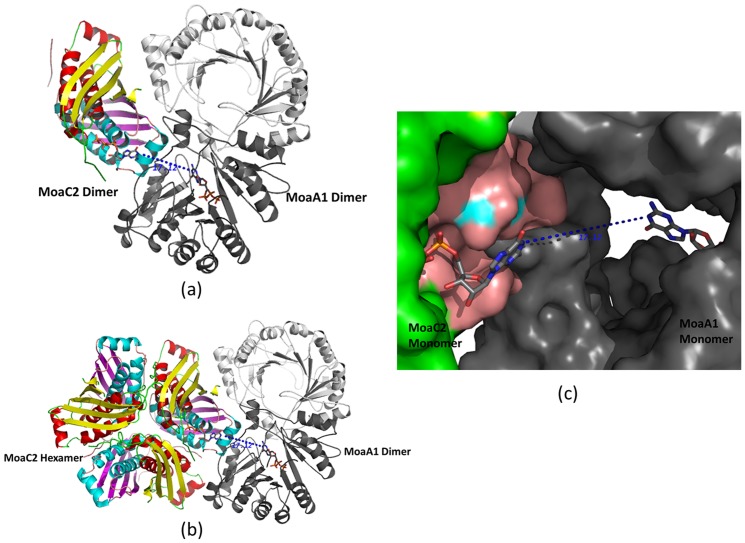
Schematic cartoon representation of the protein-protein docking, with the putative ligand sites identified by a GTP molecule represented as stick. Only the closest ligand sites are shown for clarity. (**a**) The docked model of MoaC2 dimer with MoaA1 dimer. (**b**) The docked model superposed on the MoaC2 hexamer. (**c**) A close up view in surface representation of the ligand binding sites of monomer A of MoaC2 and monomer X of MoaA1. The distance in Å is measured between the two guanosine rings.

Current literature studies have not shown MoaC or MoaA to exist separately as monomers. The *S. aureus* MoaA too exists as a dimer. Therefore dimers of MoaC2 and MoaA1 were given as input to the *ClusPro* 2.0 software. The content of the asymmetric unit of MoaC2 and the model of MoaA1 were taken as the ligand and receptor respectively for docking studies and the output structures analyzed visually. Upon visual examination, approximately 2/3rds of the models show reasonable interaction between the dimers. Based on the interactions, most of these output models can be broadly grouped into a couple of modes. In the first mode that is seen in the majority of the models (∼18), the interaction occurs mainly through the α2 helix and the subsequent loop of the first monomer and and the α1 helix) of the second monomer of MoaC2 with the C-terminus of both monomers of MoaA1, although one monomer is the dominant interacting partner with MoaC2. In another mode that is observed less frequently (∼6), the dimeric interactions occurs mainly through the α1 helix, the loop residues 101–105 and residues 142–150 of the first monomer and Arg46, Glu103 and Asp150 of the second monomer (MoaC2) with the C-terminii of both the MoaA1 monomers (Figure 8a). In this mode, most of the hydrogen bonds are formed between A monomer of MoaC2 with both monomers of MoaA1 (X and Y). It has been reported in human homologs that C-terminal of MoaA1 interacts with MoaC2 [15] and this is in qualitative agreement in both the modes of the docked model. Although this mode is found less frequently than the first, this mode is qualitatively better as both electrostatic and hydrophobic interactions contribute to this mode and it is also compatible with higher order oligomer (hexamer) whereas the other mode is predominantly electrostatic. Hence, this mode was considered for further analysis.

The interacting residues of the docked MoaC2 and MoaA1 complex were identified by Protein Interactions calculator (*PIC*) web server (http://pic.mbu.iisc.ernet.in/; [25] as well by visually examining the docked models and are given in Table S3. The protein complex is stabilized by a variety of H-bonds, ionic and hydrophobic interactions and is consistent with the predictions of PPI-*Pred*.

Having seen that the dimers of MoaA1 and MoaC2 can form a complex, we went on to see if this docked model is compatible with other known oligomeric states. As mentioned earlier, the functional oligomeric state of both MoaA and MoaC are as yet unknown. While MoaA1 has been shown to exist as a dimer in solution, MoaC2 can exist as a dimer, though it is most stable as a hexamer. This hexameric association is conserved in all the available structures of MoaC from different organisms, irrespective of the crystallization condition, crystal symmetry, etc. The docked MoaA1: MoaC2 model was then superposed on to the hexameric assembly of MoaC2 and is shown in Figure 8b. As can be seen, there are no steric clashes between the docked MoaA1 dimer and the four other monomers of MoaC2.

Significantly, the docking has brought the ligand binding pockets of one monomer of MoaA1 and MoaC2 in close proximity. The ligand binding region of the X monomer of MoaA1 is approximately 17 Å (as measured between the guanosine rings) from the ligand binding site of the A monomer of MoaC2. Figure 8c shows a surface representation of this region, with the ligands (GTP) superposed to their respective binding sites. As can be seen from Figure 8c, the product of the first enzyme (MoaA1) can access the adjacent binding site of MoaC2, to carry out the subsequent step in the Moco synthesis pathway. This apparent fusion of the two binding sites might also provide a basis that why GTP has the second highest docking score. Which clearly implies that why GTP was bound loosely in *T. thermophilus* structure. It is important to note that no information was provided to the docking server regarding the binding sites. In fact, both MoaA1 and MoaC2 dimers were docked in their *apo* forms. Still, the apparent ‘fusion’ of the functional sites, as well as the compatibility with known oligomeric states of MoaC2, lends credence to the fact that these two proteins might indeed form a complex during precursor Z formation.

## Conclusions

The crystal structure of the Moco-biosynthesis protein MoaC2 (Rv0864) in its *apo* form from *Mycobacterium tuberculosis* has been determined to 2.2 Å. The protein adopts a modified form of the ferredoxin fold, with an additional α helix. Analysis of this structure and comparison with the other homologs in the PDB show that 31 residues are conserved and these residues have a functional or a structural role.

Lys78, which is conserved in all the MoaC proteins is located in the α2 helix of the adjacent dimer form three strong hydrogen bond from its NZ atom (Average distance 2.8 Å) with the main chain carbonyl group of residues Ser81, Pro85 and Cys87 which results in an approximately three fold symmetrical arrangement. The α1 helix 48–57, which is not typically present in the ferredoxin fold, shows a higher deviation when superposed with the other existing reported structures and it is not typically present in ferredoxin like fold. This helix lies adjacent to the ligand binding site and may impart functional role. Though the functional oligomeric state of the protein is still unknown, surface accessibility calculations suggest that it is stable both as a dimer and hexamer. This hexameric association is seen in available MoaC structures, suggesting that this association is important.

The structure of Rv0864 was solved in its *apo* form. Persistent efforts to crystallize the protein in the presence of GTP (co-crystallization as well as crystal soaking) did not result in a GTP- bound structure. A review of literature suggested that two different pathways were plausible for the formation of precursor Z from GTP. The molecular-docking studies of the probable ligands with the protein suggested that PBM has the highest docking energy followed by GTP, suggesting PBM as the more likely ligand. PBM also has an additional H-bond, as compared to GTP. This study has also shown that one of the proposed pathways (2b) can be eliminated as not energetically favourable.

The result of sequence based interaction study using *STRING* 9.0 helped in identifying the interacting partner of MoaC2. Once MoaA1 was identified as the interacting partner, the Protein Protein Interaction-Pred server was used to predict the interacting residues. This server too confirmed the interaction between the two proteins and identified patches on both proteins as interacting residues. In the absence of structure of *M.tuberculosis* MoaA1, a model was built using a homologous structure from the PDB as a template. The two structures were then docked to better understand the interaction between MoaC2 and MoaA1. The interaction between dimers- is possible, as dimers of both MoaC2 and MoaA1 are stable in solution [15]. The dimer-dimer interaction between MoaC2 and MoaA1 occurs mainly through a helix and a loop of MoaC2 with the C-terminal of both monomers of MoaA1. More importantly, the docking has resulted in creating a channel from the binding sites of MoaA1 and MoaC2 that is conducive for the transfer of the product generated by MoaA1 to become the substrate for MoaC2. Further experimental work has been initiated in one (AA) of our laboratories.

## Materials and Methods

### Data collection and data statistics

The cloning, overexpression, purification, crystallization, data collection and structure solution of the protein has been described previously [3]. While refinement of this structure at 2.5 Å was in progress, another data set from a crystal grown from an identical crystallization condition as before was collected at Beamline BM14, ESRF, Grenoble. This crystal diffracted to 2.20 Å with an exposure time of 11 sec, the crystal to detector distance of 207 mm and an oscillation range of 0.5°. The reflections were indexed using the *iMOSFLM* program [26] and scaled with *SCALA* [27]. The *CTRUNCATE* program [28] was used to convert intensities to structure factors. *HKL1.98.2* [29] suite was also used for processing and scaling of the data sets. *POINTLESS* [27] suggests the possible space group for this dataset as *P*2_1_3, similar to the earlier data. The only difference between the two data sets is the reduction in the cell length by 2.6 Å. The diffraction data statistics are given in Table 2.

**Table 2:** Data Collection Statistics.

Wavelength (Å)	0.97
Space Group	P2_1_3
Unit cell parameter (Å)	91.9
Resolution range (Å)	27.7-2.20 (2.28-2.20)
No. of observations	75559 (8529)
No. of unique reflection	12651 (1550)
Multiplicity	5.9 (5.5)
I/σ (I)	9 (2.5)
Completeness (%)	99.9 (99.9)
*R*_merge_ †	0.17 (58)
**Refinement statistics**	
*R*_work_	0.20
*R*_free_	0.24
**Deviation from ideal geometry**	
Bond length (Å)	0.009
Bond angle (°)	1.11
Protein Model	
No. of subunits in A.U	2
Protein Atoms	1922
Water Molecules	65
**Ramachandran plot (%)**	
Mostly favoured regions	92.1
Additional allowed regions	7.1
Generously allowed regions	0.4
Disallowed regions	0.4

† R_merge_ = Σ_hkl_Σ_i_|I_i_(hkl)−(I(hkl))|/Σ_hkl_Σ_i_ I_i_(hkl); where I_i_(hkl) is the intensity of the ith observation of reflection hkl and(I(hkl)) is the average intensity of the i observations. Values in parentheses are for the highest resolution shell.

### Structure solution, Refinement and validation

As the cell dimensions between the two datasets differ by ∼3%, the previously refined partial model was taken as the search model for, molecular-replacement calculations, done using *Phaser* [30] as implemented in the *CCP4* suite [31] which resulted in a unique solution with no steric clashes. Two copies of the search model were unambiguously placed in the asymmetric unit. The solution obtained from the MR calculation was subjected to rigid-body refinement using *PHENIX* [32] and subsequently positional refinement was performed. The crystallographic *R* factor and *R*_free_ [33] converged to values of 27% and 33% respectively and electron density maps computed. After several rounds of model building using *Coot* [34] and refinement with *REFMAC5* from *CCP4* suite, the *R* factor and *R*_free_ values converged to 20% and 24% respectively. To remove any model bias a simulated annealing omit map [32] was calculated and the resultant electron density map agreed with the model. All atoms were refined with unit occupancies. In the final refined model, the A chain, lacks 21 residues at the N-terminus as well as residues 30–34, 158–164, while in the B chain there is no interpretable electron density for the N-terminal 22 residues and residues 158–162 near the C-terminus. The final model also accommodates 65 water molecules. All the residues are in the allowed regions of the Ramachandran map [35] except one residue at the N- and C-termini of the A chain (Ala23 and Arg166) and other geometric parameters are also well within the acceptable values. The program *PROCHECK* [36] and *MOlPROBITY* [37] were used to check and validate the quality of the final refined models. Co-ordinates have been deposited in the Protein Data Bank [38] with accession code 4FDF.

### Docking studies with GTP and intermediates compound formed during the formation of precursor Z

Docking studies were performed with GTP and other probable ligands as mentioned in Kanaujia *et al*., 2010 [8] in order to estimate their binding affinities towards MoaC2 (Rv0864) using the program *AUTODOCK* 3.0.5 [39]. The Lamarckian genetic algorithm implemented in *Autodock* is a hybrid of a genetic algorithm (GA) with an adaptive local search (LS) method [40].

The 3D structures of the ligands GTP, FPT, PBT, PBM and E were built and optimized using the BUILDER module in *Insight II* [41] and *Sybyl 8.0* (Tripos Associates, Inc., St. Louis, MO). After docking, the interaction energy between the docked ligand and the receptor protein was calculated using the empirical scoring function feature of *Autodock*. Rests of the parameters were set to their default values.

### Sequence based interaction studies

In order to identify the interaction of Rv0864 (MoaC2) with other interacting partner the sequence based interaction database *STRING* (http://string-db.org/) was used. The software runs a set of prediction algorithms and transfers known interactions from model organisms to other species based on predicted orthology of the corresponding proteins [42], [43]. The software identifies potential interacting partners based on a varied set of diverse interactions, including existing information from other organisms, genetics, phylogenetic co-occurences, functional associations and the output is given in the form of a confidence score for each of the four methods.

### Protein-Protein Docking

Having identified that MoaA1 and MoaC2 can interact directly, the two proteins were docked computationally using the *ClusPro* 2.0 server [44]. *Mycobacterium tuberculosis* MoaA1 is 359 amino acid long and its structure is unknown. However, a *BLAST* search of the MoaA1 sequence against the PDB identified a homologue from *Staphylococcus aureus* (PDBID 2FB3) [7], with a sequence identity of 37%. A homology model of MoaA1 was generated with the *S. aureus* model as the template using the *SwissProt* server and validated using standard tools.

To predict the structure of a complex, *Cluspro* 2.0 requires only the atomic coordinates of the two molecules and outputs forty docked models based on electrostatic, hydrophobic, Van der Wall-electrostatic and balanced interactions (10 docked conformations for each type of interaction). Although the models are ranked by a scoring function, the authors recommend that these solutions be analysed visually to decide its feasibility. The interactions observed in these docked conformations were visually examined using the software *PyMol* (v.1.2r3pre; Schrodinger LLC) and also using the Protein Protein Interface Prediction Server PPI*-Pred* (http://bmbpcu36.leeds.ac.uk/ppi_pred/; [24] *and* PIC web server (http://pic.mbu.iisc.ernet.in/) [25].

### Structural analysis

Structures were superposed using the program *STAMP* [45]. It uses multiple sequence alignment using the amino acid sequence information followed by an initial superposition of structures. Accessible surface area, number of contacts and temperature factor was calculated using the program *Areaimol*, *Contact* and *Baverage* [31]. The sequence alignment was produced using the program *ClustalW* [46] and the figure was generated using the program *ESPript* 2.2 [47]. The *PISA web server* (http://www.ebi.ac.uk/msd-srv/prot_int/cgi-bin/piserver; [19] was used to know the biological assembly and to calculate accessible surface area and buried surface area of protein.

## Supporting Information

Table S1 **Root mean square deviation in α1 region of Rv0864 (MoaC2) with other structure.**
(DOC)

Table S2 **List of hydrogen bonds between source atom (MoaC2) and different ligands were given and docking energy of different ligands with Rv0864 (MoaC2) was given in kcal/mol.**
(DOC)

Table S3 **Hydrogen bonding in between MoaC2 and MoaA1 of Model 2.**
(DOC)
