# Correction: Structural Insights into Putative Molybdenum Cofactor Biosynthesis Protein C (MoaC2) from *Mycobacterium tuberculosis H37Rv*

**DOI:** 10.1371/annotation/49d4d96b-fafa-43cc-8c98-6146816656ed

**Published:** 2013-06-21

**Authors:** Vijay Kumar Srivastava, Shubra Srivastava, Ashish Arora, J. Venkatesh Pratap

The name of the second author was spelled incorrectly. The correct name is: Shubhra Srivastava.

