# Supplementary material for: Structural Insights into Putative Molybdenum Cofactor Biosynthesis Protein C (MoaC2) from Mycobacterium tuberculosis H37Rv
Source: PLoS One. 2013 Mar 19;8(3):e58333. doi: 10.1371/journal.pone.0058333 (PMC3602415; doi:10.1371/journal.pone.0058333)
Supplement: Table S1 — Root mean square deviation in α1 region of Rv0864 (MoaC2) with other structure. (DOC) [file pone.0058333.s001.doc]

**Supplementary Table S1**

Root mean square deviation in α1 region of Rv0864 (MoaC2) with other structure.

| PDBID | %Sequence identity | Root mean square deviation |
| --- | --- | --- |
| 1EKR | 21.43 | 0.919 |
| 3JQM | 15.38 | 1.199 |
| 2EEY | 7.14 | 1.909 |
| 2EKN | 28.54 | 0.436 |
| 2OHD | 7.14 | 0.476 |
