# Supplementary material for: Structural Insights into Putative Molybdenum Cofactor Biosynthesis Protein C (MoaC2) from Mycobacterium tuberculosis H37Rv
Source: PLoS One. 2013 Mar 19;8(3):e58333. doi: 10.1371/journal.pone.0058333 (PMC3602415; doi:10.1371/journal.pone.0058333)
Supplement: Table S2 — List of hydrogen bonds between source atom (MoaC2) and different ligands were given and docking energy of different ligands with Rv0864 (MoaC2) was given in kcal/mol. (DOC) [file pone.0058333.s002.doc]

**Supplementary Table S2**

List of hydrogen bonds between source atom (MoaC2) and different ligands were given and docking energy of different ligands with Rv0864 (MoaC2) was given in kcal/mol.

| **SOURCE ATOM** | **GTP**  (-5.48 kcal/mol) | **FPT**  (-4.05 kcal/mol) | **PBT**  (-3.13  kcal/mol) | **PBM**  (-6.36 kcal/mol) | **E**  (-4.36 kcal/mol) |
| --- | --- | --- | --- | --- | --- |
| NZ LYS A62 | O1B (3.20 Å) | O20 (3.22 Å)  O28 (3.46 Å)  O30 (3.22 Å) | O20 (3.07 Å) | O25 (2.59 Å) | - |
| ND1 HIS B88  NE2 HIS B88 | O3G (2.29 Å)  - | O30 (3.16 Å)  O31 (2.25 Å) | O28 (2.75 Å)  O29 (2.89 Å) | O28 (2.09 Å)  - | O20 (3.41 Å)  - |
| OD1 ASP A137  OD2 ASP A137  O ASP A137 | O1G (3.46 Å)  O1G (3.08 Å) | -  -  - | O26 (3.20 Å)  O26 (3.10 Å)  - | O25 (3.32 Å)  -  O32 (3.42 Å) | -  O11 (3.06 Å)  O12 (3.27 Å) |
| NZ LYS A140  N LYS A140 | PA (3.31 Å)  - | O20 (2.72 Å)  - | O25 (3.06Å), O22 (3.08 Å) | O31 (2.53 Å)  O32 (2.63 Å) | O22 (2.58 Å)  O22 (2.53 Å) |
