# Supplementary material for: Structural Insights into Putative Molybdenum Cofactor Biosynthesis Protein C (MoaC2) from Mycobacterium tuberculosis H37Rv
Source: PLoS One. 2013 Mar 19;8(3):e58333. doi: 10.1371/journal.pone.0058333 (PMC3602415; doi:10.1371/journal.pone.0058333)
Supplement: Table S3 — Hydrogen bonding in between MoaC2 and MoaA1 of Model 2. (DOC) [file pone.0058333.s003.doc]

**Supplementary Table S3**

Hydrogen bonding in between MoaC2 and MoaA1 of Model 2.

| **MoaC2 Residues** | **MoaA1 Residues** | **Distance (Å)** | **MoaC2 Residues** | **MoaA1 Residues** | **Distance (Å)** |
| --- | --- | --- | --- | --- | --- |
| NH1 ARG  B46 | **OE2 GLU Y314** | 2.74 | NE2 GLN B103 | **OD1 ASP Y310** | 3.10 |
| NH2 ARG  B46 | **OE2 GLU Y314** | 3.43 | NE2 GLN B103 | **OD1 ASP Y310** | 3.18 |
| NH2 ARG  B46 | **OE1 GLU Y314** | 2.75 | OD1 ASP A105 | NH1 ARG Y 21 | 2.80 |
| OG SER  A48 | N ARG Y23 | 3.47 | OD2 ASP A105 | NH1 ARG Y 21 | 2.95 |
| N SER  A48 | O ARG Y21 | 3.31 | **O VAL A 142** | NH2 ARG X 331 | 3.19 |
| NE2 GLN A50 | **SG CYS X290** | 3.59 | OD1 ASP A 143 | NH1 ARG Y 23 | 2.74 |
| NE2 GLN A50 | OD1 ASP X325 | 3.23 | OD2 ASP A 143 | NH1 ARG Y 23 | 3.37 |
| OE1 GLN A50 | NE2 GLN X330 | 2.83 | OD2 ASP A 143 | NH2 ARG Y 23 | 2.71 |
| NE2 GLN A50 | **SG CYS X290** | 3.59 | **OD1 ASP A 149** | NH2 ARG Y 321 | 2.77 |
| NE2 GLN A50 | OD1 ASP X325 | 3.23 | **OD2 ASP A 149** | NH2 ARG Y 321 | 2.74 |
| OE1 GLN A50 | NE2 GLN X330 | 2.83 | **OD2 ASP A 149** | NE ARG Y 321 | 2.91 |
| N ALA  A53 | OG1 THR X324 | 3.0 | **OD1 ASP A 150** | NH2 ARG Y 322 | 2.72 |
| O VAL A101 | NH1 ARG X21 | 2.61 | **OD2 ASP A 150** | NH2 ARG Y 322 | 2.80 |
| O VAL A101 | NH2 ARG X21 | 2.84 | **OD2 ASP A 150** | NE ARG Y 322 | 2.91 |
| NE2 GLN A103 | O ARG X 21 | 2.78 | **OD1 ASP B 150** | NH1 ARG Y 321 | 3.03 |
| OEI GLN A103 | **SG CYS X22** | 3.77 | **OD2 ASP B 150** | NH1 ARG Y 321 | 2.82 |
